# The Efficacy and Safety of Shen Guo Lao Nian Granule for Common Cold of Qi-Deficiency Syndrome: Study Protocol for a Randomized, Double-Blind, Placebo-Controlled, Multicenter, Phase II Clinical Trial

**DOI:** 10.1155/2017/1806461

**Published:** 2017-09-24

**Authors:** Xuemei Liu, Juanjuan Fu, Tao Fan, Wei Liu, Hongli Jiang, Ruiming Zhang, Hong Ding, Haimiao Yang, Siyuan Hu, Yuhong Huang, Guanhong Li, Ying Lan, Bin She, Bing Mao

**Affiliations:** ^1^Department of Integrated Traditional and Western Medicine, West China Hospital, Sichuan University, Chengdu 610041, China; ^2^Department of National Good Clinical Practice, The Affiliated Hospital, Chengdu University of Traditional Chinese Medicine, Chengdu 610041, China; ^3^Department of Respiratory Medicine, The Affiliated Hospital, Changchun University of Traditional Chinese Medicine, Changchun 130051, China; ^4^Department of Good Clinical Practice, The First Affiliated Hospital, Tianjin University of Traditional Chinese Medicine, Tianjin 300193, China; ^5^Center of Good Clinical Practice, The Second Affiliated Hospital, Tianjin University of Traditional Chinese Medicine, Tianjin 300150, China; ^6^College of Acupuncture and Tuina, Traditional Chinese Medicine of Chengdu University, Chengdu 610041, China

## Abstract

**Background:**

Common cold is one of the most frequently occurring illnesses in primary healthcare services and represents considerable disease burden. Common cold of Qi-deficiency syndrome (CCQDS) is an important but less addressed traditional Chinese medicine (TCM) pattern. We designed a protocol to explore the efficacy, safety, and optimal dose of Shen Guo Lao Nian Granule (SGLNG) for treating CCQDS.

**Methods/Design:**

This is a multicenter, randomized, double-blind, placebo-controlled, phase II clinical trial. A total of 240 eligible patients will be recruited from five centers. Patients are randomly assigned to high-dose group, middle-dose group, low-dose group, or control group in a 1 : 1 : 1 : 1 ratio. All drugs are required to be taken 3 times daily for 5 days with a 5-day follow-up period. Primary outcomes are duration of all symptoms, total score reduction on Jackson's scale, and TCM symptoms scale. Secondary outcomes include every single TCM symptom duration and score reduction, TCM main symptoms disappearance rate, curative effects, and comparison between Jackson's scale and TCM symptom scale.

**Ethics and Trial Registration:**

This study protocol was approved by the Ethics Committee of Clinical Trials and Biomedicine of West China Hospital of Sichuan University (number IRB-2014-12) and registered with the Chinese Clinical Trial Registry (ChiCTR-IPR-15006349).

## 1. Introduction

Common cold is one of the most frequently occurring illness present in primary healthcare services [1] and is the prototype of upper respiratory tract infection (URTI). Colds are usually caused by viral infection; rhinoviruses cause 30% to 50% of colds throughout the year and 80% of colds during peak season, and up to 200 other viruses are implicated [2]. The main symptoms are sore throat, rhinitis, rhinorrhoea, cough, and malaise [1, 3, 4]. These symptoms typically peak at 1–3 days and last 7–10 days but can persist for several weeks [3, 4]. Although common cold is self-limiting and majority of the symptoms can improve spontaneously within weeks, due to its high prevalence [3] and severe impact on a group of susceptible individuals such as children, elderly, poor sleepers [5], and patients with chronic respiratory diseases [6], it still represents a considerable societal burden associated with absence from school or work, doctor visits [7], and huge medical costs, which have been estimated to be 17 billion dollars directly and 25 billion dollars indirectly in the US per year [8].

Recent evidence has demonstrated the clinical benefits and safety of complementary and alternative medicine (CAM) for common cold [9], and traditional Chinese medicine (TCM) plays an important role in CAM. TCM is a 3000-year-old holistic system of medicine that combines medical herbs, acupuncture, food therapy, massage, and therapeutic exercise for both extensive treatment and prevention of diseases [10]. The effects of TCM on common cold have been demonstrated in a large number of clinical trials and systematic reviews in which TCM were shown to relieve symptoms and shorten the course of common cold [11–14].

Treatment based on syndrome differentiation is the character and spirit of TCM. Along with wind-cold, wind-heat, and summer-heat dampness patterns, Qi-deficiency syndrome is one of the most frequent TCM pattern in common cold [15]. Caused by insufficiency of essential Qi, manifested as fatigue, short of breath, and spontaneous perspiration, people with chronic illness, serious illness, or overworked, who can easily be attacked by exterior evil, are prone to Qi-deficiency syndrome in common cold. The invading evil may disturb the ascending and descending function of lung and spleen Qi, causing fatigue, cough, and running nose. What is worse, without sufficient essential Qi, it is difficult to dispel evil out quickly, resulting in a longer disease duration [15]. There are a number of clinical trials which assessed TCM formula for common cold with either wind-cold, wind-heat, or summer-heat dampness syndrome [16–18]; however, few studies have addressed the effects of TCM on Qi-deficiency syndrome which is an important but easily neglected syndrome pattern in common cold.

Shen Guo Lao Nian Granule (SGLNG) is based on an empirical formula of TCM for common cold of Qi-deficiency syndrome (CCQDS) and has been approved by the State Food and Drug Administration of China for a clinical trial (Certified Number: 2003L01916). It is composed of Platycarya strobilacea Sieb. et Zucc, Polygonum cuspidatum Sieb. et Zucc, Bidens pilosa L., Dendranthema indicum, E. sinica Stapf, Siraitia grosvenorii, Panax quinquefolius, Lentinus edodes, and Astragalus membranaceus Bunge (Table 1).

Unpublished pharmacodynamic studies made a rat model of CCQDS by sulphur dioxide, smoke, and cold air stimulation [19, 20]. Guided by clinical index equivalent transformation method, respiratory frequency and load swimming time of rats were carefully observed, while low respiratory rate represents short of breath in human with CCQDS, and short load swimming time means fatigue [21]. Data revealed that SGLNG can reduce cough frequency and respiratory rate and extend load swimming time. Additionally, in an in vitro study, 500 mg/mL and 1000 mg/mL of SGLNG significantly inhibited the replication of influenza virus RP8 in embryonated eggs in a simultaneous inoculation and infection model, and it also inactivated adenovirus at a concentration of 250 to 500 mg/mL. In vivo, SGLNG alleviated pathological changes of viral pneumonia in mice and had protective effects on* Haemophilus influenzae* and* Staphylococcus aureus*-induced lung inflammation. Additionally, SGLNG can reduce endotoxin-induced fever in rabbits. There was no chronic toxicity found in rats administered different doses of SGLNG (50x, 15x, and 5x normal dose) for 3 months except that blood glucose was higher in the 50x and 15x groups. Therefore, SGLNG have a significant inactivation and inhibition on respiratory tract virus infection.

A multicenter, randomized, active controlled clinical study was conducted from year 2005 to year 2007 to investigate the efficacy of SGLNG in treating common cold of Qi-deficiency syndrome (CCQDS) in patients aged between 45 and 75 years. Results showed that patients in the SGLNG group exhibited a greater reduction in symptom total score and relieving single symptoms like fatigue, muscle, and joint soreness, with no adverse event reported, compared to a classic TCM formula of Yin Qiao Jie Du Granule [22]. All of this reveals a trend for treating CCQDS by SGLNG. However, due to the disadvantages of positive-controlled trial and lacking of large samples, the exact efficacy of SGLNG in treating CCQDS and the effective dose are still unknown.

Therefore, we designed this randomized, double-blind, placebo-controlled, multicenter, phase II clinical trial to investigate the efficacy of SGLNG in treating CCQDS with the outcome measures focused on symptom reduction and to detect the optimal therapeutic dosage.

## 2. Methods/Design

### 2.1. Design

**Figure 1:**
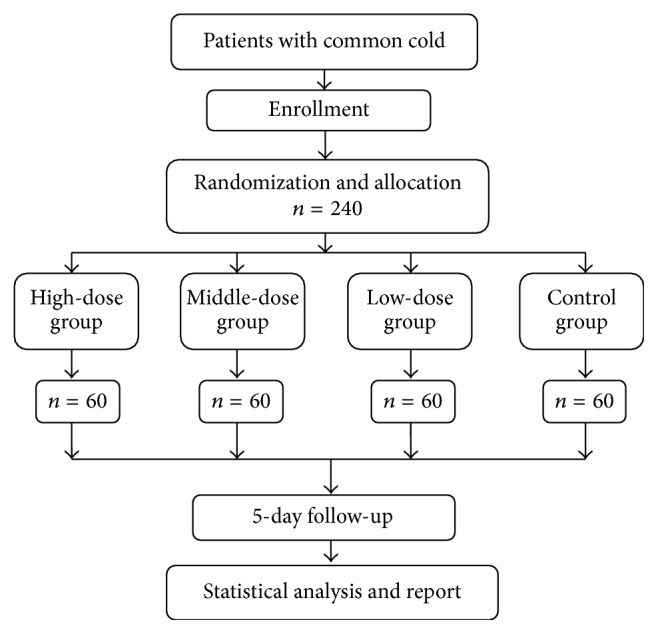
Flowchart. 240 patients will be recruited in the study and randomly allocated to one of four groups (high-dose group = 60; middle-dose group = 60; low-dose group = 60; control group = 60). After 5 days of treatment, we analyze the data collected and make conclusion.

This is a multicenter, randomized, double-blind, placebo-controlled, phase II clinical study (Figure 1) adhering to the recommendations of the “CONSORT statement” [23] and the “SPIRIT statement” [24]. The study is financially supported by Hebei Baishan Pharmaceutical Co. Ltd., Hebei, China, which has played no role and will play no role in the study design, analysis, data interpretation, or decision to submit results.

#### 2.1.1. Medicinal Preparations

The main components of SGLNG are shown in Table 1. One gram of SGLNG equals 2.22 g raw plant drugs. Each component of SGLNG is produced by soaking the material in distilled water for 30 minutes, boiling it in 10 volumes of water (v/w) for 1 hour, extracting it with water twice, filtering, and condensing it to the concentration of 1 g/ml and processing it to a fine granular form by spray drying [25]. Considering the characteristics of Chinese herbal medicine, the placebo is prepared by 10-fold dilution of the study drug (SGLNG) and then condensed into granules, which are composed of 10% SGLNG and 90% maltodextrin, and the colour is made identical to SGLNG by adding artificial pigment (tartrazine, sunset yellow, and sucralose). After a 10-fold dilution, the study drug exhibits very little clinical or pharmacological effects and therefore can be used as a placebo [26]. This preparation of placebo has been used in previous studies [16]. SGLNG and placebo are nearly identical in appearance, taste, and scent and dissolve quickly in water. The production and packaging of SGLNG as well as placebo granules are provided by the sponsor unit (Hebei Baishan Pharmaceutical Co. Ltd., Hebei, China). The granules are produced in a single batch (SGLNG batch number Y140501; Placebo batch number Y140501-1). Based on the high performance liquid chromatography techniques, and good manufacturing practices (GMPs) full-range management, this company can guarantee the consistency of granule composition. All drugs are concealed in uniform packages with the same labels except drug numbers, and three bags of the test drug (the dose for one drink) are packed in a small box, with each large box containing 5 plus 1-day dosage (a total of 18 small boxes inside). Participants are isolated from each other after enrollment and are advised to take the drugs at home to avoid chatting about the drugs.

#### 2.1.2. Subjects Recruitment

We advertise the study using advertisements and posters placed on billboards in the participating hospitals and communities. Potential participants are encouraged to visit the study offices immediately when they experience cold symptoms. Participants are recruited from the out-patient departments and emergency rooms of five participating centers: West China Hospital of Sichuan University, the Affiliated Hospital of Chengdu University of TCM, the Affiliated Hospital of Changchun University of TCM, the first Affiliated Hospital of Tianjin University of TCM, and the Second Affiliated Hospital of Tianjin University of TCM. West China Hospital is the leading unit of the five centers. These centers are defined primarily geographically. All patients are required to provide their written informed consent prior to study entry.

Enrolled participants are randomly allocated to one of four groups (high-dose group, middle-dose group, low-dose group, and control group) and undergo a 5-day treatment and a 5-day follow-up (Table 3). 

#### 2.1.3. Staff Training

In each participating center, a chief investigator is appointed to be responsible for the whole clinical process (subject recruitment, intervention, follow-up, and data collection), adhering to the study protocol. All personnel involved in this trial are trained prior to trial initiation.

#### 2.1.4. Ethics and Trial Registration

Ethical approval was obtained from the Ethics Committee of Clinical Trials and Biomedicine of West China Hospital of Sichuan University (number IRB-2014-12), and the other four centers received ethical approval from local ethics committees. The study is conducted in accordance with the Declaration of Helsinki and registered with the Chinese Clinical Trial Registry (registration number: ChiCTR-IPR-15006349).

### 2.2. Objects

#### 2.2.1. Diagnostic Criteria

*Diagnostic Criteria of Western Medicine. *Participants must fulfill the following criteria [18]: (1) answering “yes” to either “do you think you have a cold?” or “do you think you are coming down with a cold?”; (2) reporting at least 1 of 4 cold symptoms: nasal discharge (runny nose), nasal obstruction (plugged or congested), sneezing, or sore (scratchy) throat; (3) scoring at least 2 points on Jackson's scale [27].

*Diagnostic Criteria of TCM. *The diagnostic criteria for common cold of Qi-deficiency syndrome (CCQDS) are based on the Guidelines for Clinical Research of New Chinese Medicine [28] (Table 2). Main symptoms include aversion to cold, stuffy, running nose with sneezing, fatigue, and shortness of breath. Secondary symptoms include fever, sore throat, headache, cough, expectoration, spontaneous perspiration, pale or red tongue, white or thin yellow tongue coat, and pulse conditions (float or weak pulse). To be diagnosed with CCQDS, patient must fulfill all symptoms listed in (1) (in Table 2) and at least one symptom listed in (2) (in Table 2) of the main symptoms, and three or more symptoms in secondary symptoms, combined with tongue and pulse conditions.

#### 2.2.2. Inclusion Criteria

Inclusion criteria are that participants are aged between 45 and 75 years; have symptoms of Qi-deficiency in normal times such as fatiguing easily, poor appetite, or suffering more frequent colds (≥6 times/year) when seasons and climates change; meet the diagnostic criteria of a common cold within 48 hours of onset; meet the diagnostic criteria of CCQDS; and possess an understanding of the trial procedure and a willingness to sign the consent form.

Although the name of the medication indicates that its possible target population might be the “elderly people,” based on the TCM pathology of common cold with Qi-deficiency, patients with younger age than the elderly patients may also present CCQDS. Therefore, in the RCT to assess the clinical efficacy of SGLNG, we extend the age of the study population from 45 to 75. This may provide broader target population for the medication and also provide evidence for phase III study in the future.

#### 2.2.3. Exclusion Criteria

The participants are not eligible to participate in the trial if they meet any of the following criteria: (1) have acute viral pharyngitis and laryngitis, acute herpes angina, acute conjunctivitis pharynx, or acute tonsillitis, suspected influenza, and vaccination against influenza within 21 days before enrollment; (2) history of severe cardiovascular, pulmonary, renal or haematopoietic system disease, immunodeficiency disease (infection with human immunodeficiency virus, etc.), diabetes, and a previous abnormal glucose tolerance test or a significantly abnormal ECG test; (3) liver function test (alanine transferase (ALT), aspartate transferase (AST), total bilirubin (TBIL), albumin (ALB), alkaline phosphatase (ALP), and *γ*-glutamyltranspetidase (*γ*-GT)) >1.5 times compared to the upper limit of normal reference values, or abnormal serum creatinine (Cr); (4) use of any medication intended to treat common cold, which may affect the study results; (5) body temperature > 38.5°C; (6) pregnancy or potential pregnancy or lactation; (7) hypersensitivity or allergy to any component of the test drug; (8) current mentally or legally disabled; (9) current or previous admission to other clinical trials within 3 months before enrollment; (10) symptom of paroxysmal sneezing, nasal congestion, or a runny nose are considered allergic diseases by investigators.

To distinguish a cold event from an allergy, the participants who are allergic to or possess contraindications for the use of certain components of the study drugs will be excluded at enrollment. Additionally, all the clinical researchers involved in the study are educated on the difference between common cold and an allergic event and informed to distinguish between these events during enrollment, such that “cold symptoms occur one at a time, generally last for 5–7 days, with yellow/greenish nasal discharge, and may or may not be accompanied by a fever, with slight body aches and pains, but allergic symptoms occur rapidly and all at once and last as long as the allergy-causing agent is present, with clear and watery nasal discharge, not associated with a fever, and with no body aches or pains” [29]. The clinical researchers will also be informed to enquire about the allergic history of each participant and to ask whether the participant has had contact with the allergic components recently.

#### 2.2.4. Withdrawal and Dropout

The study participation will be terminated if the amount of study medication taken is poor (actual usage of trial drug <80% or =120%) or the participant is unable to cooperate with the trial investigators and/or unable to complete the follow-up. When an allergic reaction or a serious adverse event is revealed, a participant is unwilling to continue the trial, or symptoms do not improve or worsen after taking the study drugs for 72 hours, the patient will be withdrawn from the trial after a researcher's assessment, and the data will be considered invalid but will be included in the full analysis set (FAS). If the participant drops out, he or she will be contacted via phone interviews, e-mail, or written letters. The reason for study discontinuation and the last time the medication was taken will be recorded. Participants who exit the trial due to allergies, poor curative effects, or other adverse events will receive appropriate treatment.

### 2.3. Sample Size Calculation

The sample size was determined using Encyclopaedia Medical Statistics 3.2 software (PEMS 3.2, Guangzhou Jing far Pharmaceutical Research Co., Ltd., Guangzhou, China) with 90% power and a significance level of 0.05 (two-sided). According to Lizogub et al. [30], the sample size should consist of 50 participants in each group. Considering the potential 20% loss, 60 participants are needed for each group. As there is a low-dose group, a middle-dose group, a high-dose group, and a control group with five centers involved, a total of 240 participants will be recruited in this study, with 48 patients recruited at each center.

### 2.4. Randomization and Blinding

According to a stratified block randomization method, the PRCO PLAN function of the analysis system of SAS software (SAS Institute, Inc., Cary, NC, USA) was used to generate the random number grouping table according to the number of cases and the random proportion of the participating units. There are 5 centers and 4 groups (high-dose group, middle-dose group, low-dose group, and control group), and each group is divided into two layers in a 1 : 1 ratio, while one layer is aged between 45 and 60 years and the other layer is aged between 61 and 75 years. So each group in one single center will recruit 12 patients, with 6 patients between 45 and 60 years old and 6 patients between 61 and 75 years old. Eligible patients are randomly allocated to the high-dose group, middle-dose group, low-dose group, or control group in a 1 : 1 : 1 : 1 ratio. The randomization sequence was generated by the Health Statistics Department of the Fourth Military Medical University of China, which is independent of the trial. The details of randomization sequences are unknown to researchers and outcome assessors who participate in the study and kept in two copies in an opaque, sealed, and stapled envelope that will be kept concealed until the end of the outcome assessment.

The blinding codes are preserved and protected separately by West China Hospital and the other four participating centers. Emergency letters are prepared by a statistician in which the random code and group assignment are included. If the blind code is exposed, the corresponding patient will be excluded. Any of the following situations could call for a blind breaking and urgent measure: a serious adverse event, serious complication, or deteriorative condition.

Following the blinding principle, the five participating centers are isolated from the sponsor unit during the study and they will contact only the supervision agency (Beijing Chuangli Medical Technology Development Co., Ltd., China), which is responsible for contacting participating centers and monitoring the progress of research but is blind to group assignment.

### 2.5. Interventions

#### 2.5.1. Interventions

Patients who meet the inclusion criteria and none of the exclusion criteria are randomized into one of four treatment groups: (1) high-dose group: 3 bags of SGLNG/time; (2) middle-dose group: 2 bags of SGLNG plus 1 bag of placebo granule/time; (3) low-dose group: 1 bag of SGLNG plus 2 bags of placebo granule/time; and (4) control group: 3 bags of placebo granule/time.

Each participant receives a large box and be instructed to dissolve one small box of the contents in approximately 200 mL of warm liquid and take orally after each meal, 3 times daily for 5 days. The participants are isolated from each other after enrollment and advised to take the drugs at home to avoid discussing the drugs. Participants, researchers, and outcome assessors will remain blinded to the treatment allocation throughout the study.

Throughout the study period, participants will not be permitted to use any other treatments for the common cold, including oral medications and TCM therapies such as cupping, massage, or acupuncture. Medications for concomitant diseases, such as hypertension, are allowed. The dosage, duration, and name of any concomitant medication or treatment will be recorded.

#### 2.5.2. Follow-Up and Compliance

The follow-up visits will last for 5 days after the treatment. Details of the study procedures are shown in Table 3. During the 11-day study period, a total of four visits (on the 1st, 4th, 6th, and 11th days) will be performed. After the enrollment, the investigators will contact each patient every day to obtain the details of dynamic changes in cold symptoms and remind them to attend the clinical visits on certain dates. The participants are required to collect the packages after taking the granules and bring them on the 4th and 6th days visits. The total actual dose is defined as the total number of bags that are actually used (by counting the number of consumed capsules).

### 2.6. Outcome Measurements

#### 2.6.1. General Information and Physical Examination

The demographics, family history, past history, medical history, vital signs, and physical characteristics (body temperature, respiration rate, heart rate, blood pressure, and body weight) are obtained and recorded on the 1st day (day 1). Physical examination and a review of concomitant medications are conducted on the 4th, 6th, and 11th days of the intervention.

#### 2.6.2. Safety Assessment

Safety assessments are taken on the 1st day (day 1) and the 6th day of the intervention including (1) vital signs and body weight; (2) full blood count, urine routine plus urinary sediment tests; (3) liver function tests (ALT, AST, TBIL, ALB, ALP, and *γ*-GT); (4) renal function tests (Cr, cystatin C, urinary albumin, and urinary NAG enzyme); (5) fasting blood glucose and 2-hour postprandial blood glucose; and (6) electrocardiogram.

The chronic toxicity test in rats revealed that SGLNG may raise blood sugar during the dosing period (lasted for 3 weeks) in both 15 times and 50 times the clinical daily dose groups; it is possible that long-term high-dose of SGLNG taken may cause blood sugar disorders in diabetic patients. The fasting blood glucose and 2-hour postprandial blood glucose tests are used to observe the effect of SGLNG on blood glucose during the trial as a safety index. What is more, it can also exclude participants with unknown diabetes before enrollment. Although the earlier multicenter, randomized, active controlled clinical study did not report that SGLNG can cause abnormal blood glucose in patients [22], we intended to add this criterion for safety consideration.

#### 2.6.3. Excluding Observation

Chest X-ray, urine pregnancy test (only for females), fasting blood glucose, and 2-hour postprandial blood glucose are assessed on the 1st day of the intervention.

#### 2.6.4. Compliance Assessment

The compliance of each participant is calculated from the total dose that was actually taken and the total dose required by the study protocol. The total actual dose is defined as the total number of bags that are actually used (by counting the number of consumed capsules), and the total required dose is defined as the number of bags that should be used during the 5-day treatment. Compliance is assessed on the 11th day after the intervention. The formula is as follows:

(1) $$\begin{array}{c} Compliance=\frac{Total\;\;actual\;\;dose}{Total\;\;required\;\;dose}\times 100\%. \end{array}$$

#### 2.6.5. Primary Outcomes

*Duration of All Symptoms*. Duration of all symptoms is defined as the number of days from enrollment to the day that all symptoms disappear (the time from the symptom severity score above zero until 2 days of the severity score marked as zero). If symptom severity is marked above zero on the day after a single day of zero, the cold is defined as continuing. If the day marked above zero is the 11th day, this is defined as the end of the cold, but if symptoms last more than 11 days, follow-up will continue via phone call or e-mail until all symptoms are absent. The first day of medication administration is defined as the 1st day (day 1) in the study. The start of the day is defined as 6:00 a.m., and symptoms absent before 6:00 a.m. are regarded as occurring the day before.

Because all the participants in the trial have Qi-deficiency syndrome and normally present certain Qi-deficiency symptoms (fatigue, shortness of breath, and spontaneous perspiration), we define the disappearance of Qi-deficiency symptoms (symptom score = 0) (Table 2) as all the symptoms returning to the basic level present before the onset of common cold.

*The Total Score Reduction of Jackson's Scale*. Jackson's scale [27], which is specific for diagnosis and symptom severity assessment of common cold, contains 8 symptoms (sneezing, headache, malaise, chilliness, nasal discharge, nasal obstruction, sore throat, and cough) and rates as 0 = absent, 1 = mild, 2 = moderate, and 3 = severe. The symptom score will be summed on the 1st day and the 4th, 6th, and 11th days after intervention, and the score reduction will be calculated between the 1st day and the days after the intervention.

The symptom severity and score are assessed by researchers who are blind to the trial, and one participant is assessed by the same researcher consistently throughout trial. Participants will not be allowed to assess their overall illness or level of severity to avoid assessment bias.

*The Total Score Reduction of TCM Symptoms Scale*. In compliance with the severity measurement of common cold in the Guidelines for Clinical Research of New Chinese Medicine [28], symptom score of CCQDS is obtained from a four-point Likert-type symptom severity scale (Table 2), with different score criteria for main symptoms and secondary symptoms: (1) main symptoms: 0 = no symptoms; 2 = mild; 4 = moderate; 6 = severe; (2) secondary symptoms: 0 = no symptoms; 1 = mild; 2 = moderate; 3 = severe. The total score of TCM symptoms (including main and secondary symptoms) will be summed on the 1st, 4th, 6th, and 11th days, and the score reduction is defined as the scores on the 1st day minus the scores after the intervention, which will be calculated on the 4th, 6th, and 11th days.

#### 2.6.6. Secondary Outcomes

*The Duration of Every Single TCM Symptom. *The duration of every single TCM symptom is defined as the number of days from enrollment to the time when each single TCM symptom resolves or returns to the level (symptoms specific for Qi-deficiency) before the onset of the cold with no recurrence within 48 hours. The duration of each TCM symptom (including main symptoms and secondary symptoms) will be recorded.

*The Disappearing Rate of TCM Main Symptoms on the 4th and 6th Days. *The four main symptoms of CCQDS include aversion to cold, stuffy and running nose with sneezing, fatigue, and shortness of breath. The disappearing rate is measured as the percentage of disappeared symptoms (number of disappeared symptoms) on the 4th and 6th days.

*The Respective Total Score Reduction of TCM Main Symptoms and Secondary Symptoms. *The scores of TCM main symptoms evaluated for severity (Table 2) will be summed on the 1st day. The total score reduction of TCM main symptoms is defined as the score on the 1st day before intervention minus the scores after the intervention, which will be calculated on the 4th, 6th, and 11th day. Similarly, the total score reduction of TCM secondary symptoms will be calculated on the 4th, 6th, and 11th day by recording the score on the 1st day before intervention minus the scores after the intervention.

*Curative Effect*. The curative effect is calculated as the percentage of cumulative symptom score reduction (PSSR) recorded on the 11th day and categorized into two grades as effective (PSSR ≥ 50%) or invalid (PSSR < 50%). According to Jackson's scale and TCM symptom scale, the curative effect will be recorded, respectively. The formula is as follows: PSSR = (symptom score before treatment − symptom score after treatment)/symptom score before treatment × 100%.

*Comparison between Jackson's Scale and TCM Symptoms Scale*. Jackson's scale of common cold and TCM symptoms scale are overlapping in terms of certain symptoms, while TCM symptoms cover some other abnormal symptoms than Jackson's scale. The effect of interventions on Jackson's scale and TCM symptom scores will be compared on the 4th, 6th, and 11th days, and the consistency between the two scales will be assessed by correlation analysis.

### 2.7. Adverse Events

Participants are required to truthfully report symptom changes. Any adverse events (such as worsening of symptoms, concomitant diseases, and abnormal laboratory results) during the test will be recorded and treated appropriately by the intervention administrators until the patient's laboratory results return to normal and symptoms disappear. Follow-up will be performed via hospital visits and phone calls according to the severity of adverse event. In cases of severe adverse events, investigators are asked to record and report the events to the Drug Registration Department of the State Food and Drug Administration, the Safety Supervision Department, the Provincial Drug Supervision Bureau, the trial applicant, and the ethics committee within 24 hours.

### 2.8. Statistical Analysis

The full analysis set (FAS) is the primary analysis set for efficacy with an intention-to-treat (ITT) principle. In the FAS, all patients will be treated with at least one dose of the study drug and at least one clinical observation will be recorded in the study. All subjects without any major protocol deviations will be involved in the per-protocol set (PPS). The safety analysis will be conducted for randomized subjects who have completed at least one study visit and have safety data. Descriptive statistics will be performed on continuous variables, frequencies, and categorical variables. All symptom durations, total score reductions on Jackson's scale and TCM symptoms, the duration of each TCM symptom, the decreasing rate of TCM main symptoms, and the reduction score of TCM symptoms will be estimated with the Kaplan-Meier technique and compared using the stratified log-rank test. Comparisons among different dose groups and age layers will be conducted with an analysis of variance (ANOVA) and a least significant difference *t*-test. In this multicenter trial, the Cochran-Mantel-Haenszel test stratified by clinical centers and the logistic regression model adjusted for covariates will be used. Statistical analyses will be conducted by an independent statistician from the Health Statistics Department of the Fourth Military Medical University using the SAS statistical software 9.1.3 (SAS, Cary, North Carolina State University, North Carolina, USA).

## 3. Discussion

There is an urgent necessity to develop an effective treatment for CCQDS due to its frequent occurrence and heavy burden. This study intend to examine the clinical efficacy and safety and find optimal dose of SGLNG for treating CCQDS, in a randomized, double-blind, and placebo-controlled trail.

We conduct this study not only to propose a new remedy for common cold, but also to practice the methodology of syndrome differentiation for clinical research in TCM. This is the first study that addresses the efficacy and safety of a TCM formula for this specific but frequently seen TCM syndrome, Qi-deficiency in common cold in a double-blind and placebo-controlled trail. We assume that this design, which is in line with TCM theory and also typical of individualized and phenotype-based medicine, will contribute to more convincing results and robust evidence for the efficacy of study medications and cause new insight into common cold treating. Apart from the syndrome differentiation-based selection of the study population, there is another strength in this study. We perform both Western and TCM scales to measure the effects of the study medication. They are overlapping in terms of certain symptoms, while TCM symptoms cover some other abnormal symptoms than Jackson's scale, but the relationship between them has not been explored before. In this study, the scores of Jackson's scale and TCM symptom scale are compared at each time point, so as to detect their merit and weak point, respectively, which may provide more powerful evidence for further measurements of common cold. To our knowledge, this is the first trial to evaluate the difference between Jackson's scale and TCM symptom scale.

There are some limitations in this study. Due to limited budget, we do not perform culture assessing for inclusion of patients with common cold, which may cause mistakenly recruiting patients with potential influenza. To minimize this mistake, all the clinical researchers involved in the study are educated on the difference between common cold and influenza. Patients who experience severe symptoms will be excluded at enrollment, while those with symptoms which were slight at first but worsened rapidly during the study will be advised for culture assessing in clinical or even dropout from the trail. Apart from that, we employ Jackson's scale for the outcome measurement, while other latest questionnaires such as the Wisconsin Upper Respiratory Symptom Survey [31] would be useful. However, we did not include this questionnaire due to the unavailability of a Chinese version. Additionally, the study trail does not cover the mechanisms underlying CCQDS and potential pathways through which TCM formula can treat the disease in this specific population, which still require further detection.

In summary, by rigorous methodology for a clinical trial and syndrome differentiation-based approach, the results of this study will provide robust clinical evidence regarding the efficacy, safety, and optimal dose of SGLNG for treating CCQDS.

## 4. Conclusion

This trial asks if SGLNG reduces CCQDS symptoms and which is the optimal dose.

## Figures and Tables

**Table 1:** Formulation and dose of SGLNG.

Chinese Pinyin name	English name	Latin name	Source	Weight (%)^*∗*^
Hua Xiang Shu Guo Xu	Infructescence of Platycarya strobilacea Sieb.	Platycarya strobilacea sieb.et Zucc	The dried infructescence of *Platycarya strobilacea* Sieb.	14.96
Hu Zhang	Polygonum cuspidatum	Polygonum cuspidatum Sieb.et Zucc	The dried rhizome and root of Polygonum cuspidatum	11.97
Gui Zhen Cao	Bidens bipinnata	Bidens pilosa L.	The dried stems and leaves of *Bidens bipinnata*	14.96
Ye Ju Hua	Mother Chrysanthemum	Dendranthema indicum	The dried flowers of Mother Chrysanthemum	11.97
Ma Huang	Ephedra	E. sinica Stapf	The dried herbaceous stem of Ephedra	5.98
Luo Hang Guo	Siraitia	Siraitia grosvenorii	The dried fruit of *Siraitia*	14.96
Xi Yang Shen	American ginseng	Panax quinquefolius	The dried root of American ginseng	2.99
Xiang Gu	Mushrooms	Lentinus edodes	The dried mushrooms without roots	11.97
Huang Qi	Astragalus membranaceus	Astragalus membranaceus Bunge	The dried stems and leaves of Astragalus membranaceus	9.97

^*∗*^The weight of every single herb in each bag of SGLNG (5 g).

**Table 2:** TCM symptoms of common cold and symptom score.

		Symptom^*∗*^	None = 0	Mild = 2	Moderate = 4	Severe = 6
Main symptom	(1)	Aversion to cold	None	Slight aversion to cold, do not need to add cloth	Aversion to cold, need to add cloth	Aversion to cold, need to add thick cloth, or bedding
		Stuffy and running nose	None	Mild nasal congestion, runny nose occasionally, not affecting respiration	Nasal congestion, runny nose, affecting breathing	Obvious nasal congestion, running nose, mouth breathing
	(2)	Fatigue	None	Lack of energy, not resistant to labor, (slight heavier than usual)	Mental weariness, slightly affecting daily activities (heavier than usual)	Limb weakness, cannot support daily activities (much heavier than usual)
		Short of breath	None	Shortness of breath after labor (slight heavier than usual)	Shortness of breath after slight labor (heavier than usual)	Obvious shortness of breath when resting (much heavier than usual)

		Symptom	None = 0	Mild = 1	Moderate = 2	Severe = 3

Secondary symptom		Fever	None	37.3–37.5°C	37.6–37.9°C	≧38.0°C
		Sore throat	None	Dry throat with slight pain	Significant with obvious pain when swallowing	Sore and swollen throat with severe pain
		Headache	None	Slight and occasionally	Severe and lasting	Severe and affect working
		Cough	None	Occasionally in daytime, not affecting daily life	Occasionally day and night, slightly affecting sleep	Frequent cough day and night, affecting rest and sleep
		Expectoration	None	Stick sputum, not easy to cough out	Stick or yellow sputum	Stick or yellow sputum with large volume
		Spontaneous perspiration	None	Yes (severe than usual)	—	—
		Tongue and pulse condition^#^	Tongue color	Pale □ Red □		Others
			Tongue coat	Thin white □ Thin yellow □		Others

^*∗*^Scores represent the severity of symptoms. ^#^The score of tongue and pulse will not be recorded.

**Table 3:** Study schedule for patients. On the 1st day, researchers collect the data of medical and treatment history, efficacy observation, safety assessment, and exclusive observation of patients, as well as randomization, allocation, and drug distribution. On the 4th day (fulfilled for 4 days to the first time of medicine consume), patients take the second visit for physical examination, and researchers record symptoms scores, combined medication, and adverse event (if any). The 6th ± 1 day (fulfilled for 6 days to the first time of medicine consume), patients take the third visit for physical examination and the second safety assessment, and researchers record symptoms scores, combined medication, adverse event (if any), and recycled medicines and pack box, and drug quantity statistics. On the 11th ± 2 day (fulfilled for 11 days to the first time of medicine consume), patients take the fourth visit for physical examination, and researchers record symptoms scores, combined medication, and adverse event (if any).

Stage	Visit period			
Treatment	Visit 1	Visit 2	Visit 3	Visit 4
Day	1 day	4th day	6th ± 1 day	11th ± 2 days

*Basic medical history recording*				
Informed consent signing	×			
General information recording	×			
Medical and treatment history recording	×			
Complication and symptoms recording	×			
Physical examining	×	×	×	×
Combined medication recording		×	×	×
*Efficacy observation *				
Symptoms scores recording	×	×	×	×
*Safety assessment *				
Routine blood test	×		×	
Routine urine + urinary sediment microscopy	×		×	
Liver function test	×		×	
Renal function test	×		×	
Fasting blood glucose + 2 h postprandial blood glucose	×		×	
Electrocardiogram	×		×	
*Excluding observation *				
Chest X-ray	×			
Urine pregnancy test (female)	×			
Fasting blood glucose + 2 h postprandial blood glucose	×			
*Adverse events observation *				
Adverse event recording		×	×	×
*Other works *				
Randomization and allocation	×			
Drug distribution	×			
Medicines and packing box recycling			×	
Drug quantity statistics			×	

## Abbreviations

ALB: Albumin
ALP: Alkaline phosphatase
ALT: Alanine transferase
ANOVA: Analysis of variance
AST: Aspartate transferase
CAM: Complementary and alternative medicine
CCQDS: Common cold with Qi-deficiency syndrome
Cr: Creatinine
CRF: Case report form
ECG: Electrocardiogram
FAS: Full analysis set
GMP: Good manufacturing practices
ITT: Intention-to-treat
PPS: Per-protocol set
PSSR: Percentage of cumulative TCM symptom score reduction
*γ*-GT: *γ*-Glutamyltranspetidase
SFDA: State Food and Drug Administration
SGLNG: Shen Guo Lao Nian Granule
TBIL: Total bilirubin
TCM: Traditional Chinese medicine
URTI: Upper respiratory tract infection
USA: United States of America.
